# Dyspnea and the electromyographic activity of inspiratory muscles during weaning from mechanical ventilation

**DOI:** 10.1186/s13613-022-01025-5

**Published:** 2022-06-10

**Authors:** Côme Bureau, Martin Dres, Elise Morawiec, Julien Mayaux, Julie Delemazure, Thomas Similowski, Alexandre Demoule

**Affiliations:** 1 Neurophysiologie Respiratoire Expérimentale et Clinique, Sorbonne Université, INSERM, UMRS1158, 75005 Paris, France; 2 Service de Médecine Intensive et Réanimation (Département R3S), AP-HP, Groupe Hospitalier Universitaire APHP-Sorbonne Université, Site Pitié-Salpêtrière, 75013 Paris, France; 3 Département R3S, AP-HP, Groupe Hospitalier Universitaire APHP-Sorbonne Université, Site Pitié-Salpêtrière, 75013 Paris, France

**Keywords:** Mechanical ventilation, Dyspnea, Clinical study, Physiology

## Abstract

**Rationale:**

Dyspnea, a key symptom of acute respiratory failure, is not among the criteria for spontaneous breathing trial (SBT) failure. Here, we sought (1) to determine whether dyspnea is a reliable failure criterion for SBT failure; (2) to quantify the relationship between dyspnea and the respective electromyographic activity of the diaphragm (EMGdi), the parasternal (EMGpa) and the *Alae nasi* (EMGan).

**Methods:**

Mechanically ventilated patients undergoing an SBT were included. Dyspnea intensity was measured by the Dyspnea-Visual Analogic Scale (Dyspnea-VAS) at the initiation and end of the SBT. During the 30-min SBT or until SBT failure, the EMGdi was continuously measured with a multi-electrode nasogastric catheter and the EMGan and EMGpa with surface electrodes.

**Results:**

Thirty-one patients were included, SAPS 2 (median [interquartile range]) 53 (37‒74), mechanically ventilated for 6 (3‒10) days. Seventeen patients (45%) failed the SBT. The increase in Dyspnea-VAS along the SBT was higher in patients who failed (6 [4‒8] cm) than in those who passed (0 [0‒1] cm, *p* = 0.01). The area under the receiver operating characteristics curve for Dyspnea-VAS was 0.909 (0.786–1.032). The increase in Dyspnea-VAS was significantly correlated to the increase in EMGan (Rho = 0.42 [0.04‒0.70], *p* < 0.05), but not to the increase in EMGpa (Rho = − 0.121 [− 0.495 to − 0.290], *p* = 0.555) and EMGdi (Rho = − 0.26 [− 0.68 to 0.28], *p* = 0.289).

**Conclusion:**

Dyspnea is a reliable criterion of SBT failure, suggesting that Dyspnea-VAS could be used as a monitoring tool of the SBT. In addition, dyspnea seems to be more closely related to the electromyographic activity of the *Alae nasi* than of the diaphragm.

**Supplementary Information:**

The online version contains supplementary material available at 10.1186/s13613-022-01025-5.

## Introduction

In the intensive care unit (ICU), weaning from mechanical ventilation follows a well-described two-step assessment. First, readiness-to-wean criteria should be searched for, at least on a daily basis [1]. Second, once these criteria are present, a spontaneous breathing trial (SBT) is performed to evaluate whether the patient can breathe without assistance from the ventilator [1]. The SBT is deemed a failure if the patient develops clinical signs considered as reflecting respiratory distress, which are considered to relate to an imbalance between respiratory system loading and respiratory muscle capacity [1–4]. These signs of acute respiratory failure and subsequent SBT failure are deemed objective (polypnea > 35/min, PaO_2_ ≤ 50–60 mmHg with FiO_2_ ≥ 50% or SpO_2_ < 90%, increase between the initiation and end of the SBT in PaCO_2_ > 8 mmHg, tachycardia > 140/min) and subjective (signs considered as reflecting respiratory distress, increased accessory muscle activity, agitation and anxiety) [1]. If the SBT is successful, criteria for extubation are searched for. The current procedure applied within a classic population of intensive care patients is associated with 70% or more weaning success in randomized or observational studies [5, 6].

Although dyspnea is a key symptom of acute respiratory failure, dyspnea is not listed among the criteria for SBT failure and is often not even collected and therefore ignored during the SBT. Yet, dyspnea is a self-reported symptom that is easy to collect and to quantify in communicative patients, and could be used as a criterion of SBT failure [7, 8]. Dyspnea occurs when the inadequacy between the outflowing neural drive and the corresponding instreaming afferent return increases [9], which occurs in the event of respiratory system load capacity imbalance. It is of note that the mechanism through which increased respiratory drive increases dyspnea has not been fully established. In particular, it is not clear whether dyspnea is more closely linked to the central output to the diaphragm or to extradiaphragmatic inspiratory muscles [8, 9].

Here, we assessed dyspnea during an SBT. Our first objective was to determine whether dyspnea change is a reliable criterion of weaning failure and to compare dyspnea to other criteria for weaning failure to determine whether dyspnea collection should be part of the follow-up of all SBTs in an effort to improve the current MV weaning procedure. Our second objective was to establish the relationship between dyspnea and the respective electromyographic activity (EMG) of the diaphragm and two extradiaphragmatic inspiratory muscles, namely the parasternal intercostal and the *Alae nasi*.

### Patients and methods

This observational, single-center prospective study was conducted over a 1-year period in the 10-bed medical intensive care unit (ICU) of the respiratory and ICU Division of La Pitié-Salpêtrière Hospital, Paris, France.

#### Patients

The study was approved by the Comité de Protection des Personnes Ile de France VI (No. 84-14). Informed consent was obtained from patients.

Patients were eligible as soon as they met the following criteria: (1) intubation and mechanical ventilation for more than 24 h, and (2) the predefined readiness-to-wean criteria, adapted from the 2007 international consensus conference [1], on daily screening and the physician in charge decided to initiate an SBT [1]. Predefined readiness-to-wean criteria were the following: resolution of the acute phase of the disease for which the patient had received invasive mechanical ventilation, low bronchial congestion, adequate cough, adequate oxygenation defined by SpO_2_ > 90% with FiO_2_ ≤ 40% and positive end-expiratory pressure (PEEP) ≤  8 cm H_2_O, respiratory rate ≤ 40 breathes/min, a Ramsay sedation scale < 4 and stable cardiovascular status (heart rate ≤ 120 beats/min, systolic arterial blood pressure ≤ 180 mmHg and no or minimal vasopressors (norepinephrine < 5 μg/kg/min). Patients < 18 years, with neuromuscular diseases, those for whom life support would be withheld or withdrawn and patients for whom the repeated assessment of dyspnea was likely to be difficult (auditory or visual impairment, insufficient command of French, known prior cognitive or psychiatric disorder, ICU delirium) were not included in the study.

#### Study protocol

All patients were ventilated using a Servo-i ventilator (Maquet Critical Care, Solna, Sweden) equipped with the standard commercial version of the NAVA module.

The standard nasogastric feeding tube was removed and replaced by an electromyography activity of the diaphragm catheter consisting of a 16-French gastric tube equipped with electrodes.

As soon as the patients met the readiness-to-wean criteria, a 30-min SBT was performed with the patient connected to the ventilator with a pressure support level of 7 cm H_2_O and zero end-expiratory pressure. The FiO_2_ was adjusted during the SBT so as to obtain an SpO_2_ of between 90 and 95%. Electromyographic recording was performed during the whole time of the SBT. The endotracheal tube was suctioned before starting recording.

Based on the criteria adapted from the 2007 international consensus conference [1], the SBT was considered a failure if at least one the following criteria was present: (1) SaO_2_ < 90% with FiO_2_ ≥ 50%; (2) acute respiratory distress (RR ≥ 40/min, agitation, cyanosis); (3) systolic arterial blood pressure ≥ 180 mmHg; (4) sudden cardiac arrhythmias, and (5) respiratory acidosis (pH < 7.32 with PaCO_2_ ≥ 50 mmHg. If none of the above failure criteria was present, the SBT was considered successfully completed and extubation was considered. The patient was reconnected if signs of intolerance (see above) were present. The decision as to whether the SBT was passed or failed was based on the clinical judgment of the physician in charge of the patient, who was independent of the investigators.

#### Data acquisition

Anthropometric data and medical history were collected at inclusion.

##### Clinical variables

Heart rate, systemic blood pressure and dyspnea were collected at the initiation and end of the SBT. Presence of neuromuscular weakness, defined by MRC score < 48, was collected. Richmond Agitation Sedation Scale and Pain-Visual Analogue Scale (from 1 to 100 mm) were collected during the assessment that was the closest of the SBT. Medications received by the patient before and during the SBT were collected. Patients were considered able to consistently self-report dyspnea if the Dyspnea-Visual Analogue Scale (Dyspnea-VAS) variation did not exceed 1 cm for three consecutive measures [10, 11]. Dyspnea was quantified by placing a cursor on a 10 cm Dyspnea-Visual Analogue Scale (Dyspnea-VAS) bounded on the left by “no respiratory discomfort” and on the right by “worst imaginable respiratory discomfort”.

##### Airway pressure and flow

Airway flow was measured with a pneumotachograph (Hans Rudolph, Kansas City, USA) inserted between the Y-piece and the endotracheal tube before being connected to a differential pressure transducer (Validyne, Northridge, USA). Airway pressure was measured at the Y-piece by a differential pressure transducer (Validyne, Northridge, USA).

##### Electromyography of extradiaphragmatic inspiratory muscles

The EMG signal was collected by self-adhesive surface electrodes such as those commonly used to record the electrocardiogram signal in critically ill patients, with an interval of 2 cm between the two electrodes. Electrode positions varied according to the muscle being recorded. For the EMG of the parasternal intercostal muscles, electrodes were placed next to the second intercostal space as close as possible to the sternum. For the EMG of the *Alae nasi* muscles, electrodes were placed on the lateral surfaces of the nose (nostrils) [9]. EMG signals were amplified, sampled at a frequency of 10 kHz, and finally filtered between 40 and 500 Hz (PowerLab, ADInstruments, Dunedin, New Zealand). The signal was processed using the Labchart Peak Analysis module MLS380/8 (ADInstruments, Dunedin, New Zealand), which generates a root mean square of the EMG smoothed over 1-s fixed windows.

##### Electromyography of the diaphragm

The EMG of the diaphragm was obtained from the multi-electrode nasogastric NAVA catheter, an esophageal probe that collects the EMG signal with bipolar electrodes in sequential order, after checking the correct positioning of the probe and the quality of the signal. An automated processing technique tracks the displacement of the diaphragm [12]. A cross-correlation technique (for every second pair of electrodes) determines the position of the diaphragm along the array every 16 ms. Subtraction of opposite phase signals above and below the diaphragm results in a new signal: the “double-subtracted” signal. This double-subtracted signal is free from distance filtering. In addition, the signal-to-noise ratio is enhanced [12]. Diaphragm EMG was acquired at 100 Hz from the ventilator connected to a computer using commercially available software (Servo-i RCR, version 3.6.2, Maquet Critical Care), which generates a root mean square of the EMG. The root mean square of this signal is then calculated every 16 ms, and added to the RMS of the center signal [13, 14]. The resultant RMS values for every 16-ms sample can be graphically connected [15]. The RMS is linearly related to the number of motor units recruited and their firing rate. Finally, the electrocardiographic signal is detected and replaced by a value predicted from the previous diaphragm EMG value. To further reduce electrical noise disturbances and motion artifacts and to minimize electrocardiographic and electrical activity from the esophagus, the signal is eventually filtered with a cascade of filters.

##### Arterial blood gases

Blood gases were sampled at the initiation and end of the SBT or earlier in case of intolerance using an arterial catheter.

#### Signal processing

Respiratory rate (RR) and tidal volume (*V*t) were determined on a breath-by-breath basis and averaged for the first and last 5 min of the SBT recording (Labchart 7.3^®^ software, ADInstruments, Dunedin, New Zealand).

Maximum EMG (EMGmax) and the area under the curve of the EMG during inspiratory time (EMGauc) integrated from baseline to peak were calculated on a breath-by-breath basis. The first and last 5 min of the SBT were eventually averaged. Due to the variability of skin impedance [16, 17] and the patient’s morphology, EMGmax and EMGauc values were normalized to EMG activity at initiation of the SBT.

#### Statistical analysis

Statistical analysis was performed with Prism 8.0 software (GraphPad Software, USA). Quantitative variables were described by their median and interquartile range. Changes in dyspnea, breathing pattern, hemodynamics and blood gas are expressed as the difference in variables between the initiation and end of the SBT. Change in EMG activity is expressed as the ratio of the EMG activity and the end of SBT to the EMG activity at the initiation of the SBT. Contribution of extradiaphragmatic recruitment to diaphragm activity is expressed as the ratio of the EMG activity of the *Alae Nasi* to the EMG activity of the diaphragm. Categorical variables were expressed as absolute values and percentages. All analyses were performed with a type I error of 5%. Results were compared between the success and failure groups. Differences between groups were assessed with the Mann–Whitney test for continuous variables and the *χ*^2^ tests for categorical variables. Correlations between variables were evaluated using the Spearman rank correlation coefficient. receiver operating characteristic (ROC) curves were performed to evaluate the performance of indices to detect SBT failure.

On the basis of previous studies that our team has conducted on this subject and data from the literature, which estimate the probability of failure to wean at 40% [18], we predict that a population of 30 patients would achieve a sufficient number of patients who fail SBT to analyze the variables that detect SBT failure. Because we anticipated missing data on the assessment of dyspnea and poor EMG quality in some patients, we increased the sample size to 50 patients.

### Results

One hundred and forty-five patients with invasive mechanical ventilation for more than 24 h and eligible for SBT were admitted during the study period. Two patients were less than 18 years old, three had auditory impairment, three had insufficient command of French, 26 refused to participate, and, a technical reason (equipment failure, weekend) could not allow inclusion in 61.

Fifty patients were included in the study, of whom five were excluded because dyspnea could not be assessed reliably and 14 because the EMG signal was of poor quality and could not be process or interpreted. Overall, 31 patients were enrolled, of whom 14 (45%) failed the SBT; among them, three were due to cardiac failure.

Patient characteristics are shown in Table 1. Age was 65 (61–71) years and SAPS II at ICU admission was 53 (37–74). Patients were mechanically ventilated for 6 (3–10) days. The reason for intubation was acute respiratory failure for 71%, coma for 13%, cardiac arrest for 6% and shock for 6%. All patients intubated due to coma passed their SBT. Other characteristics did not differ between patients who passed and those who failed the SBT. No patient was receiving sedation at the time of SBT. Prior to SBT, patients had received midazolam (74%), and propofol (90%). One patient (weaning success group) was received continuous morphine chlorhydrate intravenous infusion during SBT and a second one (weaning success group) was receiving discontinuous morphine intravenous administration. The other patients did not receive morphine during SBT. In the days preceding the SBT, 87% of patients had received sufentanyl.

**Table 1:** Characteristics of the study population

	All patients *n* = 31	SBT success *n* = 17	SBT failure *n* = 14	*p* value
Gender, male, *n *(%)	22 (71)	12 (70)	10 (71)	1.000
Age, year	65 (61–71)	65 (51–68)	71 (63–75)	0.019
Body mass index, kg.m^−2^	25 (21–30)	26 (21–31)	24 (21–29)	0.726
Duration of mechanical ventilation prior to inclusion, days	6 (3–10)	7 (4–9)	4 (3–10)	0.509
Comorbidities				
Chronic respiratory disease, *n *(%)	7 (23%)	3 (18%)	4 (29%)	0.670
Chronic heart disease, *n *(%)	4 (13%)	1 (6%)	3 (21%)	0.304
Diabetes, *n *(%)	3 (10%)	2 (12%)	1 (7%)	1.000
Cirrhosis, *n *(%)	7 (23%)	5 (29%)	2 (14%)	0.411
Neuromuscular weakness, *n *(%)	4 (13%)	1 (6%)	3 (21%)	0.304
Reason for intubation				
Acute respiratory failure, *n *(%)	22 (71)	10 (59)	12 (86)	0.101
Coma, *n *(%)	4 (13)	4 (24)	0 (0)	0.050
Cardiac arrest, *n *(%)	2 (6)	1 (6)	1 (7)	1.000
Shock, *n *(%)	2 (6)	1 (6)	1 (7)	1.000
Severity scores				
SAPS II at ICU admission	53 (37–74)	61 (39–80)	45.5 (30–61)	0.414
SOFA at inclusion	11 (7–14)	12.5 (9.5–14)	8.5 (6–12)	1.000
Pain assessment and level of sedation				
Pain-Visual Analogic Scale, mm	0 (0–20)	0 (0–15)	0 (0–20)	0.250
RASS	0 (0–0)	0 (0–0)	0 (0–0)	0.999

Data are provided as median (interquartile range) and number (%)

*SBT* spontaneous breathing trial; *ICU* intensive care unit; *SAPS* Simplified Acute Physiology Score; *SOFA* Sepsis-related Organ Failure Assessment; *RASS* Richmond Agitation Sedation Scale

#### Dyspnea and criteria for SBT failure

Dyspnea, breathing pattern, hemodynamics and blood gas at the initiation and end of the SBT are reported in Table 2. At SBT initiation, dyspnea-VAS was no different between patients who passed the SBT and those who failed (*p* = 0.188). Systolic blood pressure (*p* = 0.233), PaCO_2_ (*p* = 0.406), pH (*p* = 0.212) and SaO_2_ (*p* = 0.462) were also no different between patients who passed the SBT and those who failed. Conversely, RR (*p* = 0.001), RR/*V*t (*p* = 0.031) and heart rate (*p* = 0.014) were higher in patients who failed the SBT than in those who passed, while PaO_2_ was lower (*p* = 0.039). At the end of the SBT, dyspnea-VAS was higher in patients who failed the SBT than in those who passed (*p* = 0.003). Respiratory rate (*p* = 0.001), heart rate (*p* = 0.001) and systolic blood pressure (*p* = 0.016) at the end of the SBT were also higher in patients who failed the SBT than in those who passed, while SaO_2_ (*p* = 0.005) was lower.

**Table 2:** Dyspnea, breathing pattern, hemodynamics and blood gas at the initiation and end of the spontaneous breathing trial

	Initiation of SBT				End of SBT			
	All patients *n* = 31	SBT success *n* = 17	SBT failure *n* = 14	*p* value	All patients *n* = 31	SBT success *n* = 17	SBT failure *n* = 14	*p* value
Dyspnea-VAS, mm	2 (0–2)	0 (0–2)	2 (2–5)	0.188	4 (0–10)	0 (0–4)	10 (8–10)	0.003
RR, min^‒1^	23 (20–28)	20 (17–23)	28 (24–31)	0.001	27 (20–35)	22 (16–25)	35 (32–40)	0.001
*V*t, ml	380 (328–476)	453 (372–557)	336 (283–388)	0.004	437 (334–551)	450 (370–563)	345 (271–513)	0.109
RR/*V*t, min^‒1^·ml^‒1^	48 (41–77)	45 (31–63)	77 (50–95)	0.031	54 (39–74)	52 (35–68)	75 (47–113)	0.625
Heart rate, min^‒1^	93 (83–102)	88 (72–105)	96 (89–102)	0.014	95 (83–110)	87 (76–101)	99 (91–114)	0.001
Systolic blood pressure, mmHg	136 (127–146)	136 (130–151)	132 (123–146)	0.233	143 (135–171)	139 (130–152)	158 (138–200)	0.016
PaO_2_, mmHg	89 (79–120)	113 (79–130)	86 (73–94)	0.039	64 (59–78)	65 (60–86)	62 (65–75)	0.218
PaCO_2_, mmHg	37 (32–46)	36 (32–42)	44 (33–52)	0.406	40 (35–45)	37 (34–41)	44 (41–66)	0.106
pH	7.45 (7.39–7.47)	7.43 (7.38–7.48)	7.45 (7.42–7.48)	0.212	7.42 (7.38–7.49)	7.42 (7.41–7.50)	7.39 (7.29–7.45)	0.150
SaO_2_, %	97 (95–100)	98 (95–100)	97 (94–98)	0.462	93 (92–95)	95 (93–99)	91 (88–93)	0.005

*SBT* spontaneous breathing trial; *Dyspnea-VAS* Dyspnea-Visual Analog Scale; *RR* respiratory rate; *Vt* tidal volume; *IBW* ideal body weight; *SaO*_*2*_ arterial saturation of oxygen

Data are provided as median (interquartile range) and number (%)

The change in dyspnea, breathing pattern, hemodynamics and blood gas between the initiation and end of the SBT is shown in Table 3. The change in dyspnea-VAS (*p* = 0.006), RR (*p* = 0.001), heart rate (*p* = 0.017), systolic blood pressure (*p* = 0.020) and SaO_2_ (*p* = 0.036) between the initiation and end of the SBT was higher in patients who failed the SBT than in those who passed. There was no difference between patients who failed the SBT and those who passed in terms of change in *V*t (*p* = 0.844), RR/*V*t (*p* = 1), PaO_2_ (*p* = 0.537), PaCO_2_ (*p* = 0.738) and pH (*p* = 0.785).

**Table 3:** Change in dyspnea, breathing pattern, hemodynamics and blood gas between the initiation and end of the spontaneous breathing trial (SBT)

	SBT success (*n* = 17)	SBT failure (*n* = 14)	*p* value
Dyspnea-VAS, mm	0 (0–1)	6 (4–8)	0.006
RR, min^‒1^	1 (− 2 to 3)	8 (4–13)	0.001
*V*t, ml	− 7 (− 74 to 80)	− 29 (− 89 to 50)	0.844
RR/*V*t	0 (− 9 to 27)	11 (− 16 to 29)	1.000
Heart rate, min^‒1^	1 (− 3 to 2)	7 (0–19)	0.017
Systolic blood pressure, mmHg	2 (− 3 to 9)	31 (5–62)	0.020
PaO_2_, mmHg	− 21 (− 40 to − 1.25)	− 21 (− 32.5 to 2)	0.910
PaCO_2_, mmHg	0 (− 2 to 4)	4 (2–12)	0.161
pH	0.02 (− 0.02 to 0.03)	− 0.07 (− 0.12 to 0.01)	0.171
SaO_2_, %	− 1 (− 3 to 0)	− 4 (− 8 to − 3)	0.036

The evolution is described by the difference in variables between the initiation and end of the spontaneous breathing trial

Data are provided as median (interquartile range) and number (%)

*Dyspnea-VAS* Dyspnea-Visual Analog Scale; *RR* respiratory rate; *Vt* tidal volume; *IBW* ideal body weight; *SaO*_*2*_ arterial saturation of oxygen

**Fig. 1:**
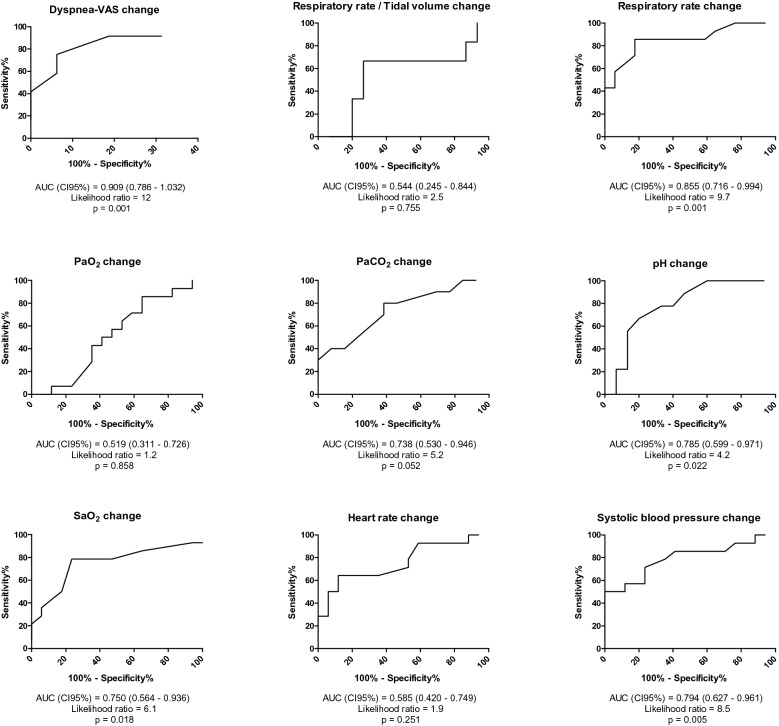
Receiver operating characteristics curve of changes in Dyspnea-Visual Analog Scale (VAS), breathing pattern, blood gas and hemodynamics to detect spontaneous breathing trial failure. Change is expressed as the difference between value at the initiation and end of the spontaneous breathing trial. *Dyspnea-VAS* Dyspnea-Visual Analog Scale; *SaO*_*2*_ arterial saturation of oxygen

Figure 1 displays the ROC curve as failure criteria for the change in dyspnea-VAS, RR/*V*t, respiratory rate, PaO_2_, PaCO_2_, pH, SaO_2_, heart rate and systolic blood pressure.

Correlation between the dyspnea-VAS and the PaO_2_, PaCO_2_, SaO_2_ and respiratory rate at the initiation and the end of spontaneous breathing trial is reported in Additional file 1: Fig. S1. Correlation between the change of dyspnea-VAS and the change in PaO_2_, PaCO_2_, SaO_2_ and respiratory rate is reported in Additional file 1: Fig. S2.

#### Dyspnea and EMG of inspiratory muscles

Changes in the EMG activity of the *Alae nasi*, parasternal intercostal muscles and diaphragm between the initiation and end of the SBT are reported in Table 4.

**Table 4:** Changes in the electromyographic activity (EMG) of the *Alae nasi*, the parasternal intercostal muscles and the diaphragm between the initiation and end of spontaneous breathing trial

	Total (*n* = 31)	Success (*n* = 17)	Failure (*n* = 14)	*p*
*Alae* *nasi*				
EMGmax	1.02 (0.94–1.25)	0.98 (0.77–1.10)	1.24 (0.94–1.60)	0.010
EMGauc	1.07 (0.66–1.44)	0.93 (0.60–1.11)	1.44 (0.91–2.40)	0.015
Parasternal intercostal muscles				
EMGmax	0.99 (0.87–1.01)	1.00 (0.98–1.01)	0.97 (0.83–1.01)	0.426
EMGauc	1.12 (0.90–1.69)	1.12 (1.04–1.25)	1.03 (0.71–1.78)	0.855
Diaphragm				
EMGmax	1.24 (0.80–2.06)	0.84 (0.60–1.57)	1.87 (1.19–2.92)	0.008
EMGauc	1.28 (0.98–1.81)	1.12 (0.79–1.70)	1.65 (1.47–2.93)	0.125
*Alae* *nasi*/diaphragm ratio				
EMGmax	0.79 (0.54–1.22)	0.87 (0.58–1.52)	0.55 (0.45–0.99)	0.039
EMGauc	0.76 (0.49–1.04)	0.72 (0.43–0.93)	0.68 (0.23–0.96)	0.812

The EMG activity is described by peak EMG (EMGmax) and area under the curve (EMGauc). Values are normalized to EMG activity at initiation of the SBT and are expressed as the ratio of the EMG activity and the end of the SBT to the EMG activity at the initiation of the SBT

Data are provided as median (interquartile range)

The change in the *Alae nasi* (*p* = 0.010 for EMGmax and *p* = 0.015 for EMGauc) and diaphragm (*p* = 0.008 for EMGmax and *p* = 0.125 for EMGauc) EMG activity between the initiation and end of the SBT was higher in patients who failed the SBT than in patients who passed. The change in parasternal intercostal EMG activity (*p* = 0.426 for EMGmax and *p* = 0.855 for EMGauc) was no different between patients who passed and those who failed the SBT. The contribution of extradiaphragmatic recruitment to the diaphragm (*p* = 0.039 for EMGmax and *p* = 0.812 for EMGauc) was lower in patients who failed the SBT than in patients who passed (Table 4). There was no correlation between extradiaphragmatic recruitment to the diaphragm and the change in dyspnea-VAS (Additional file 1: Fig. S1).

**Fig. 2:**
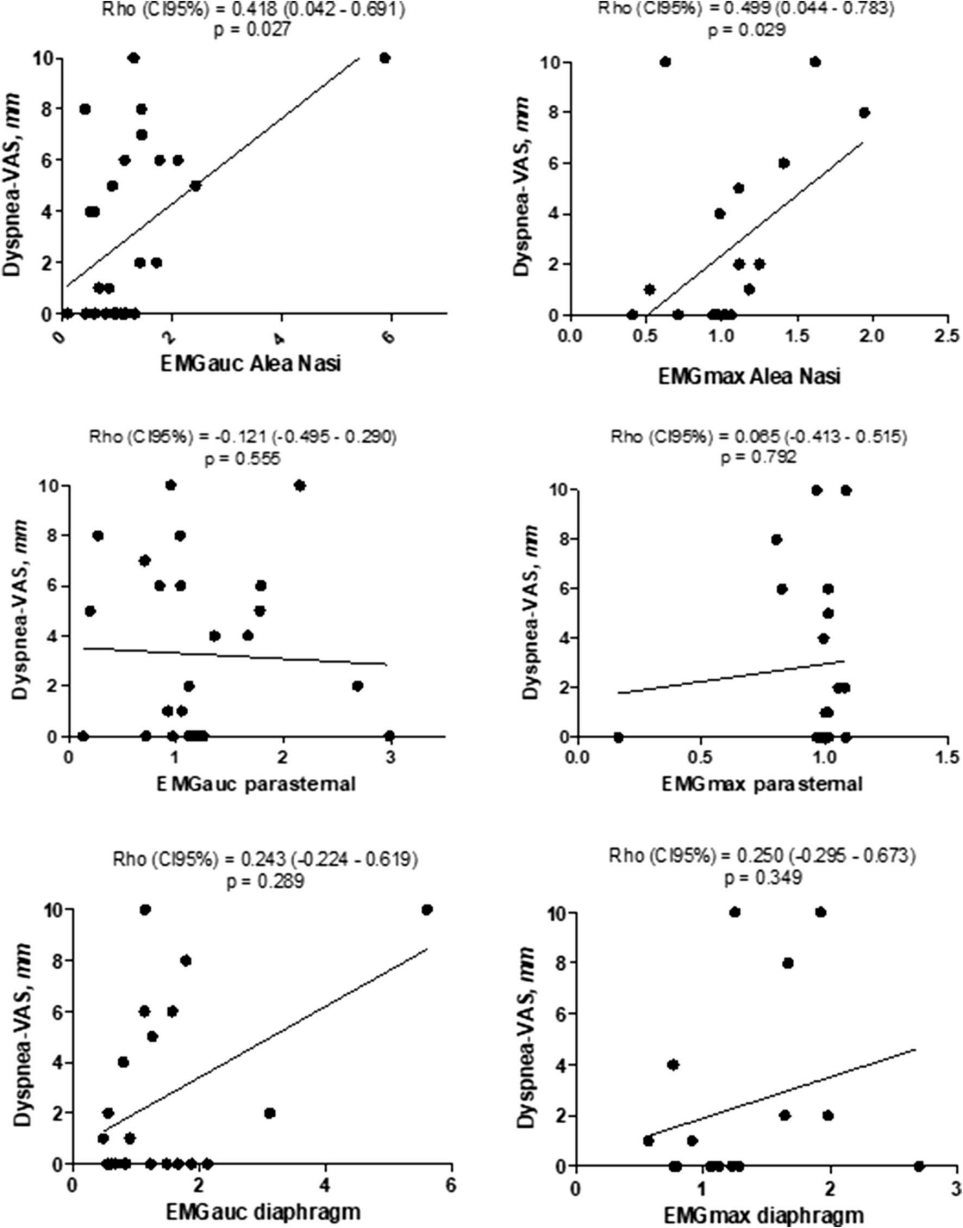
Correlation between the change of Dyspnea-Visual Analog Scale (VAS) and the change in electromyographic (EMG) activity of the *Alae nasi*, parasternal intercostal muscles and diaphragm between the initiation and end of the spontaneous breathing trial. Change in dyspnea-VAS is expressed as the difference between dyspnea-VAS at the initiation and end of the spontaneous breathing trial. The EMG activity is described by area under the curve (EMGauc) and maximum (EMGmax). Values are normalized to EMG activity at initiation of the SBT and are expressed as the ratio of the EMG activity at the end of the SBT to the EMG activity at the initiation of the SBT

There was a significant positive correlation between the change in dyspnea-VAS between the initiation and end of the SBT and the change in the *Alae nasi* EMGauc (Rho 0.418, 95% confidence interval [CI] 0.042‒0.691, *p* = 0.027) (Fig. 2). There was no significant correlation between the change in dyspnea-VAS during the SBT and the change in either the parasternal EMGauc (Rho − 0.121, CI 95% − 0.495 to 0.290, *p* = 0.555) or the diaphragm EMGauc (Rho 0.243, 95% CI − 0.224 to 0.619, *p* = 0.289). EMGmax gave similar results (Fig. 2).

There was no significant correlation between the change in *Alae nasi* EMG activity and in parasternal EMG activity (Rho − 0.016, CI 95% − 0.356 to 0.384, *p* = 0.934), between the change in *Alae nasi* EMG activity and in diaphragm EMG activity (Rho 0.308, CI 95% − 0.169 to 0.669, *p* = 0.186) and between the change in parasternal EMG activity and in diaphragm EMG activity (Rho − 0.047, CI 95% − 0.490 to 0.416, *p* = 0.845).

### Discussion

Our results can be summarized as follows. During SBT, (1) dyspnea is a reliable criterion of weaning success and failure, suggesting that dyspnea-VAS has the potential to become a useful clinical decision-making tool for SBT; (2) dyspnea seems to be more closely related to the EMG activity of the extradiaphragmatic inspiratory muscles than to the EMG activity of the diaphragm.

#### Dyspnea and neural drive to breathe

These results confirm the link between increase in respiratory drive and dyspnea. Rapid increase in respiratory loading, which happens during the SBT, in turn increases the central respiratory drive to the inspiratory muscles [8]. It has been previously observed that this increase in drive involves both the diaphragm and extradiaphragmatic inspiratory muscles [19, 20]. However, extradiaphragmatic inspiratory muscles may be strongly recruited, and even more so than the diaphragm in the case of a significant increase in respiratory loading. This is the case with sternocleidomastoid muscles in patients with chronic obstructive pulmonary disease [21, 22] and mechanically ventilated critically ill patients who fail an SBT [23] and in those who fail extubation [8]. Here, we found that the increase in EMG activity of the diaphragm and the *Alae nasi* was no different, which is consistent with a recent report that showed a similar increase in the EMG of various inspiratory muscles during a progressive decrease of pressure support in mechanically ventilated patients [20].

In our study, the increase in respiratory drive was associated with an increase in dyspnea. It is of note that dyspnea was correlated with *Alae nasi* EMG activity, but not with diaphragm EMG activity. It suggests that dyspnea is more closely linked to the central respiratory drive to extradiaphragmatic inspiratory muscles than to the central respiratory drive to the diaphragm. This result is consistent with previous reports of a significant correlation between dyspnea and extradiaphragmatic inspiratory muscle EMG activity [9, 24, 25]. This result is also consistent with a recent study showing, after extubation, a small but significant correlation between dyspnea and the ultrasound thickening fraction of the parasternal intercostal muscles but no significant correlation between dyspnea and the thickening fraction of the diaphragm [26].

In our study, the change in dyspnea was not correlated with the EMG of the parasternal muscle. If we cannot exclude that dyspnea is lesser closely linked to the central respiratory drive to the parasternal muscle than to the central respiratory drive to *Alae Nasi*, it is possible that this absence of correlation results from a lack of power or a technical issue.

#### Clinical implications: dyspnea as a monitoring tool of the SBT

Although dyspnea is a key symptom of acute respiratory failure, it is not listed among the criteria for SBT failure [1]. Our results show that dyspnea is a reliable criterion of SBT success or failure, which suggests that dyspnea-VAS could be used as a monitoring tool of the SBT rather than other variables such as heart rate, respiratory rate or arterial blood pressure. Indeed, the performance of the increase in dyspnea in detecting weaning failure seemed very good, even better than the performance of all the other variables that we tested. Our results contradict those of Haugdahl et al. who found no association between the intensity of dyspnea at the end of the SBT and the outcome of the SBT [27]. Conversely, they are compatible with those of Perren et al. who showed that, at the end of a sustained SBT, extubation success might be correlated to the patients’ subjective perception of autonomous breathing [28]. In the few studies that have investigated subjective criteria during weaning [29, 30], dyspnea was significantly associated with fatigue and patients’ lack of confidence in their ability to breathe without ventilatory support [29], whereas lack of dyspnea was identified as the best predictor of successful weaning [8, 30].

In our study, patients who failed the SBT reported an extremely high level of dyspnea. Dyspnea is a major cause of suffering for ICU patients, including anxiety; dyspnea could even contribute to the genesis of post-traumatic stress disorders [31, 32]. It must be stressed that relief of dyspnea is an essential clinical objective that, like pain, is currently considered by some authors to be a basic human right [33, 34]. Therefore, it is important to keep in mind that exposing ICU patients to these high levels of dyspnea during the SBT might not be without consequences.

The use of EMG, due to its technical difficulties of acquisition and interpretation, is not a suitable tool for monitoring SBT. In the present study, we used this tool to establish a relationship between dyspnea and the respective electromyographic activity (EMG) of the diaphragm and the two extradiaphragmatic inspiratory muscles.

#### Study limitations

This study presents limitations. First, the decision as to whether the SBT was passed or failed was based on the clinical judgment of the physician in charge of the patient; this leads to a potential inconsistency of decision criteria, even if they were based on the recommendations [1]. Second, we assessed dyspnea with only one tool, the Dyspnea-VAS. The multidimensional dyspnea profile would have provided a more detailed description of dyspnea in our patients [35]. However, this questionnaire is too long and could not be completed by patients undergoing an SBT. Third, patients were considered able to self-report dyspnea consistently if the change in the Dyspnea-VAS did not exceed 1 cm for three consecutive measurements. This led to the exclusion of several patients and limited the number of patients analyzed, but provided reliable dyspnea assessment values. Fourth, we did not measure muscle strength, only EMG as a measure of respiratory drive, and with the impossibility of making this recording reliably for several patients who had trained their exclusion. Our goal was not to assess the relationship between muscle strength and dyspnea, but rather between drive and dyspnea. Last, our work reports a longer duration of mechanical ventilation [5, 6] and a higher percentage of SBT failure [5, 6, 36] than recent studies on weaning from mechanical ventilation. Because of the small sample size and because the study was monocenter, it might be difficult to extrapolate this work. Future studies of larger sample size are needed.

#### Conclusion

In conclusion, during an SBT, change of dyspnea is more closely related to the EMG activity of the *Alae nasi* than to the EMG activity of the diaphragm. In addition, the intensity of dyspnea seems to be a reliable criterion of weaning success and failure. Subsequently, this suggests that change of dyspnea could be used as a simple monitoring tool of the SBT. The reliability of dyspnea in assessing SBT success now needs to be evaluated by a larger multicenter observational study.

## Supplementary Information

**Additional file 1**: **Figure S1.** Correlation between the dyspnea-visual analog scale (VAS) and the PaO_2_, PaCO_2_, SaO_2_ and respiratory rate at the initiation and the end of spontaneous breathing trial. **Figure S2.** Correlation between the change of dyspnea-visual analog scale (VAS) and the change in PaO_2_, PaCO_2_, SaO_2_ and respiratory rate.

## Data Availability

The datasets analyzed during the current study are available from the corresponding author on reasonable request.
