# Supplementary material for: Dyspnea and the electromyographic activity of inspiratory muscles during weaning from mechanical ventilation
Source: Ann Intensive Care. 2022 Jun 10;12:50. doi: 10.1186/s13613-022-01025-5 (PMC9187801; doi:10.1186/s13613-022-01025-5)
Supplement: Supplementary file 1 — Additional file 1: Figure S1. Correlation between the dyspnea-visual analog scale (VAS) and the PaO2, PaCO2, SaO2 and respiratory rate at the initiation and the end of spontaneous breathing trial. Figure S2. Correlation between the change of dyspnea-visual analog scale (VAS) and the change in PaO2, PaCO2, SaO2 and respiratory rate. [file 13613_2022_1025_MOESM1_ESM.docx]

**Dyspnea and the electromyographic activity of inspiratory muscles during weaning from mechanical ventilation**

Côme Bureau, MD, Martin Dres, MD, PhD, Elise Morawiec, MD, Julien Mayaux, MD, Julie Delemazure, MD, Thomas Similowski, MD, PhD, Alexandre Demoule, MD, PhD

**Figure S1. Correlation between the dyspnea-visual analog scale (VAS) and the PaO_2_, PaCO_2_, SaO_2_ and respiratory rate at the initiation and the end of spontaneous breathing trial.**

**Figure S2. Correlation between the change of dyspnea-visual analog scale (VAS) and the change in PaO_2_, PaCO_2_, SaO_2_ and respiratory rate.**

**Figure S1. Correlation between the dyspnea-visual analog scale (VAS) and the PaO_2_, PaCO_2_, SaO_2_ and respiratory rate at the initiation and the end of spontaneous breathing trial.**


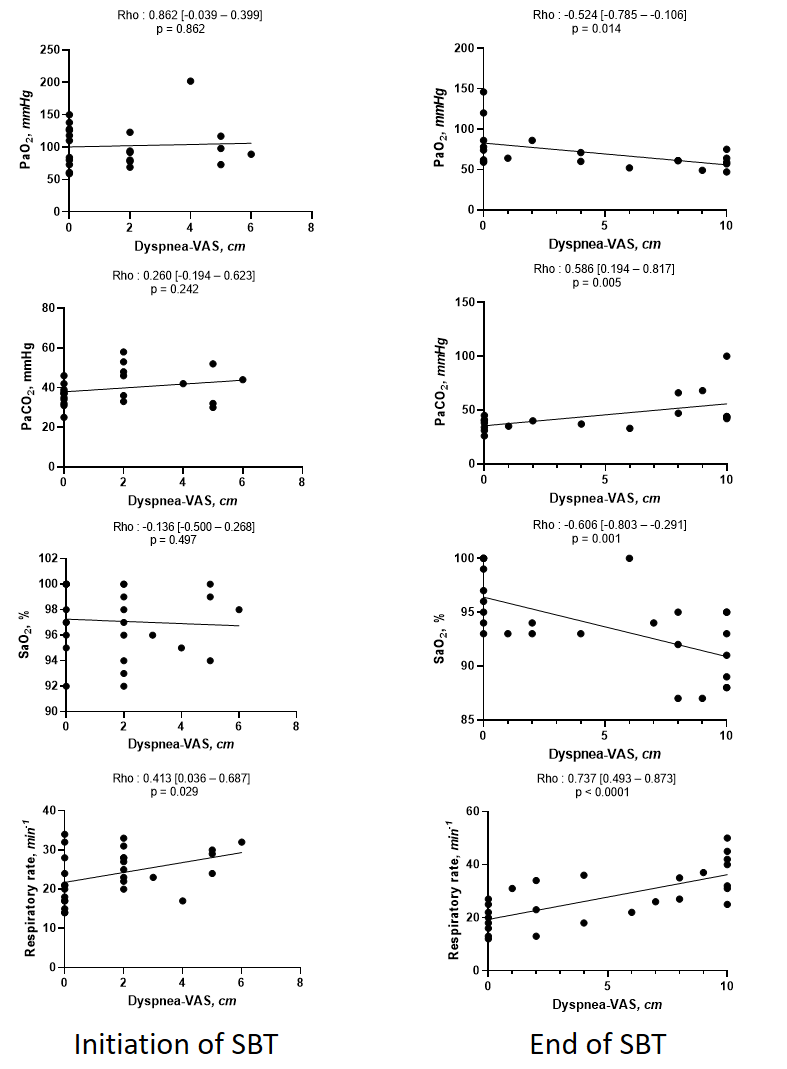


Dyspnea-VAS, dyspnea visual analog scale

**Figure S2. Correlation between the change of dyspnea-visual analog scale (VAS) and the change in PaO_2_, PaCO_2_, SaO_2_ and respiratory rate.**


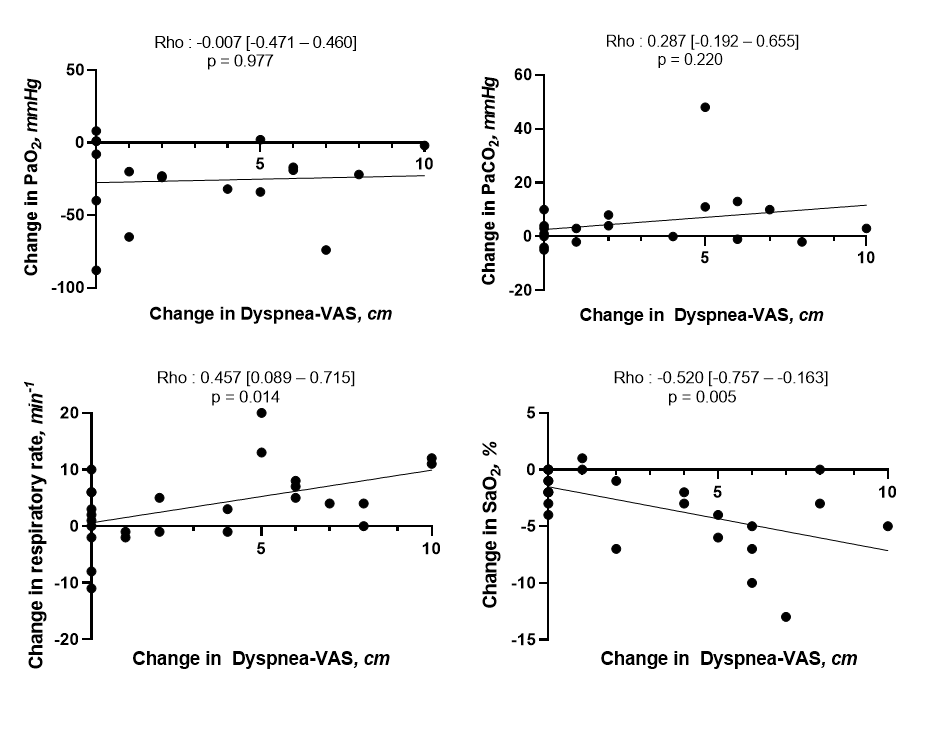


Dyspnea-VAS, dyspnea visual analog scale
